# In vitro antitumor effects of methanolic extracts of three *Ganoderema* mushrooms

**DOI:** 10.1038/s41598-025-86162-0

**Published:** 2025-01-17

**Authors:** Elshahat A. Toson, Amira A. El-Fallal, Marwa A. Oransa, Hoda M. El-Gharabawy

**Affiliations:** 1 Chemistry Department, Faculty of Science, Damietta University, New Damietta, 34517 Egypt; 2 Botany and Microbiology Department, Faculty of Science, Damietta University, New Damietta, 34517 Egypt

**Keywords:** *Ganoderma* spp., Breast cancer, Liver cancer, Cell line viability, Apoptosis, Necrosis, Biochemistry, Biological techniques, Microbiology, Applied microbiology, Fungi, Cancer, Breast cancer, Cancer prevention

## Abstract

*Ganoderma* mushrooms have a variety of pharmacological activities and may have antitumor effects. Therefore, the antitumor activity of the methanolic fruiting body extracts of three *Ganoderma* spp. will be evaluated by estimating cell viability, cell cycle parameters and the mode of cellular death. In this regard, Sulfo-rhodamine B staining and flow cytometry were used. Hepatocellular carcinoma (HepG2) and breast ductal carcinoma (T-47D) cell lines were used as cancer models, while mouse normal liver (BNL) and oral epithelial cell (OEC) lines were used as respective controls. The results revealed that *Ganoderma resinaceum* extract decreased the viability of BNL at an IC_50_ > 100 µg/mL but not that of HepG2 at an IC_50_ of 72.32 µg/mL. Additionally, *Ganoderma australe* and *Ganoderma mbrekobenum* decreased the viability of OEC cell line at an IC_50_ of 328.29 and 271.56 µg/ mL, respectively. On the other hand, the IC_50_ of T-47D were 221.95 and 236.45 µg/mL, respectively. The three extracts arrested the cell life cycle at the G1 phase in each case. *G. resinaceum* extract stimulated total apoptosis (Q2 + Q4) of 19.99% with low necrosis (Q1). However, the percentages of total cell necrosis in the T-47D cell line treated with the other two extracts were 31.10% and 18.28%, respectively while the percentages of total cell apoptosis were 6.83% and 1.78%, respectively. Thus, *G. resinaceum* significantly inhibited the viability of the HepG2 cell line, while both the *G. australe* and *G. mbrekobenum* extracts significantly decreased the viability of the T-47D cell line. These results may encourage speculation about their possible use for the therapeutic management of hepatocellular carcinoma and breast ductal carcinoma after further investigation.

## Introduction

*Ganoderma* is a significant economic genus due to its genetic diversity. It is traditional Asian medicine. In addition, *Ganoderma* species play vital roles in natural ecosystems. They are a rich source of natural antibiotics that offer a defense mechanism against several viral diseases^1^. Furthermore, they can be used as complementary medicines to alleviate the side effects of radiotherapy and chemotherapy in cancer-bearing patients^2^. Numerous reports and studies have confirmed the multidirectional biological activities of crude extracts from various *Ganoderma* spp. and of their isolated compounds. They have been proven to be antimicrobial agents^3,4^ and antimalarial agents^5^ and may also have anticancer effects^6^.

Cancer comprises a group of diseases characterized by uncontrolled cell division, replicative immortality, and cell death resistance. All of these conditions ultimately result in the formation of a mass known as a tumor. A tumor can be malignant or benign. A malignant tumor can grow and spread to other parts of the body, forming what is called the metastatic type of this disease. The benign type will grow but not spread^7^. Breast cancer is one of the most common cancers in women worldwide^8^. It is a metastatic cancer that can spread to distant organs such as the bone and liver. These factors explain its incurability^9^. Primary liver cancer (also termed hepatocellular carcinoma [HCC]). It is the fifth most common cancer in males and the seventh most common cancer in females. Furthermore, it is the third leading cause of cancer-related death worldwide^10,11^. HCC is caused by chronic liver diseases such as viral hepatitis, alcoholism, and dietary carcinogens, including aflatoxins and nitrosamines^12,13^.

The cell cycle is a highly ordered process that results in genetic information duplication and transmission from one cell generation to the next. DNA must be accurately replicated to identical chromosomal copies distributed to two daughter cells during the process^14^. Apoptosis refers to changes in the morphological features of programmed cell death. This phase is characterized by cell shrinkage, nuclear condensation and membrane blebbing. These events ultimately lead to DNA fragmentation followed by the formation of membrane bound apoptotic bodies. Eventually, these changes stimulate phagocytotic cells to engulf the apoptotic bodies formed by the affected cells. On the other hand, necrosis is used to describe early membrane damage. This results in a decrease in plasma membrane integrity and cytosolic leakage into the extracellular space. In this case, the DNA remains intact^15^.

The fruiting body, mycelium, or spore extracts of *Ganoderma* contain a wide range of bioactive molecules. These include, phenols, steroids, amino acids, lignin, mycins, vitamins, nucleosides, and nucleotides, with polysaccharides and triterpenes being the two major constituent groups^16^. They act as tumoricidal agents, potent immunomodulators and/or antioxidants. *Ganoderma lucidum* has been shown to inhibit the spread of breast cancer cells by suppressing cell adhesion, migration, invasion, and colony formation^17^. In this regard, the anticancer effect of *G. applanatum* polysaccharide (GAP) was tested in MCF-7 cells using the MTT assay. The test results showed that, the viability of MCF-7 cells was significantly decreased in a time and concentration dependent manner^18^. Furthermore, *Ganoderma* species act as chemopreventive agents^19^. The anticancer activity of *Ganoderma* polysaccharides is still thought to be closely related to their immune-stimulating effects, the cellular cytotoxicities of which are at the end stage^20^. Therefore, this study aimed to evaluate the in vitro antitumor activities of three cultivated methanolic extracts of *Ganoderma* mushrooms. The effects of these extracts on the parameters of the cell cycle, extent of cellular apoptosis and cellular necrotic rates were also investigated.

## Materials and methods

### *Ganoderma* mushroom

**Fig. 1:**
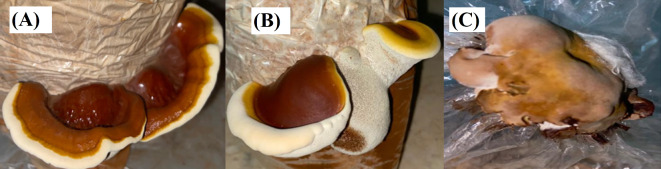
Cultivated fruiting bodies of the three *Ganoderma* species; *G. mbrekobenum* (**A**), *G. resinaceum* (**B**) and *G. australe* (**C**).

Three *Ganoderma* isolates from different host trees were used in the present study as presented in Table 1. All the isolates were maintained on potato dextrose agar media (PDA: potato extract, 200 g; glucose, 20 g; agar, 20 g and 1 L distilled water) plates and preserved on potato dextrose agar (PDA) slopes at 5 °C in a refrigerator; other stocks were preserved on 20% glycerol at -80 °C in a freezer system^21^. The mushroom cultivation process was carried out in the mushroom cultivation house at the Faculty of Science, Damietta University, during the winter and spring seasons according to the procedures reported by El-Fallal et al.^22^. The growing different *Ganoderma* mushrooms are presented in Fig. 1.

**Table 1:** Details of the different *ganoderma* strains used in the study.

No.	Ganoderma strains	GenBank code	Host (Scientific name)	Location	References
1.	*Ganoderma resinaceum EG34* *EGM*	OR590610	Casuarina tree (*Casuarina equisetifolia*)	Mansoura University, Mansoura, Egypt	^51^
2.	*Ganoderma australe W103*	OR590803	European Ash *tree* (*Fraxinus excelsior*)	Aberystwyth, Wales, United Kingdom	Not published
3.	*Ganoderma mbrekobenum EGDA G7*	LN774971	Limon tree (*Citrus Lemon*)	El-Senaniah orchards, Damietta, Egypt	^22^

### Extraction of *Ganoderma* mushrooms

First, the harvested mature mushrooms of the three *Ganoderma* species were dried in an oven at 40 °C for 8–12 h until constant weight was reached. Then, the samples were ground into fine powder using a mortar. Second, 5.0 g of each dried mushroom powder was soaked separately in 100 mL of absolute methanol for 72 h. at a room temperature of 25 °C. Whatman filter paper (no. 4) was used to filter the mixture. The residue was resoaked for 24 h. in another 100 mL of methanol. Third, the filtrate was separated, and the process was repeated three times. The filtrates from the different extraction steps were combined and completely evaporated at 40 °C to obtain the dry filtrate^23^. The weights of the obtained dry residue after the extraction process of *Ganoderma* mushrooms were 109, 850 and 184 mg for *G. resinaceum*,* G. mbrekobenum* and *G. australe*, respectively.

### Cytotoxicity assay

#### Cell culture

Hepatocellular carcinoma (HepG2) and Breast ductal carcinoma (T-47D) tumor cell lines were obtained from Nawah Scientific Inc. (Mokatam, Cairo, Egypt). Mouse normal liver (BNL) and Oral epithelial cell (OEC) were also purchased and respectively used as controls. All four cells types were maintained in DMEM media (Gibco, USA) supplemented with 100 mg/mL of streptomycin, 100 U/mL of penicillin and 10% of heat-inactivated fetal bovine serum in humidified atmosphere and in 5% (v/v) CO_2_ at 37 °C^24,25^.

#### Cells viabilities assessment using sulfo-rhodamine B (SRB) assay

Aliquots of 100 µL of cell suspension (5 × 10^3^ cells) were pipetted into 96-well plates (Tecan, Switzerland) and incubated in complete media for 24 h. The cells were treated with another aliquot of 100 µL of media containing the extracts at concentrations of 0.01, 0.1, 1, 10, 100, 300 and 1000 µg/mL. After 72 h of extracts exposure, the cells were fixed by replacing the media with 150 µL of 10% trichloroacetic acid (TCA) and incubated at 4 °C for 1 h. The TCA solution was removed, and the cells were washed 5 times with distilled water. Aliquots of 70 µL of SRB solution (4% w/v) were added, and the plates were incubated in a the dark place at room temperature for 10 min. After that, the plates were washed 3 times with 1% acetic acid and subjected to air-dryness overnight. Then, 150 µL of TRIS (10 mM) were added to dissolve the protein-bound SRB stain. The absorbance was measured at 540 nm using a BMGLABTECH^®^- FLUOstar Omega microplate reader (Ortenberg, Germany)^24,25^. The percentage of each cell viability was calculated according to the following equation:

$${\text{Cell}}\;{\text{viability}}\left( \% \right) = ~{\text{Abs}}_{{{\text{sample}}}} /{\text{Abs}}_{{{\text{control}}}} *{\text{1}}00$$

where Abs_sample_ is the absorbance of the cells treated with *Ganoderma* extracts and Abs_control_ is the absorbance of the untreated cells (control).

#### Flow cytometric assays

##### Cell cycle assessments

Aliquots of 100 µL cell line suspension (containing 5 × 10^3^ cells) either free from extracts or containning it were used in this experiment. After treatment with test extracts for 72 h, cells were collected by trypsinization and were washed twice with ice-cold PBS (pH 7.4). The cells were resuspended in two milliliters of 60% ice-cold ethanol and incubated at 4 ºC for 1 h. for fixation. Fixed cells are washed twice again with PBS (pH 7.4) and resuspended in1 mL of PBS containing 50 µg/mL RNAase A and 10 µg/mL propidium iodide (PI). After 20 min of incubation in the dark at 37 °C, cells were analyzed for their DNA contents using ACEA Novocyte™ flowcytometer, ACEA Biosciences Inc., San Diego, CA, USA, (λex/λem 535/617 nm). For each sample, 12,000 events were examined. Cell cycle distribution is calculated using ACEA NovoExpress™ software (ACEA Biosciences Inc., San Diego, CA, USA)^25–29^.

i. G0/G1-phase: This phase denotes the nonproliferating cells. Compounds with the suggested antiproliferative effect (regardless of their cytotoxicity) are expected to significantly increase this type of cell population (for example, 5-fluorouracil and methotrexate).
ii. S-phase: This phase refers to proliferating cells, particularly cells that are undergoing DNA synthesis and replication steps. Some compounds might induce S-phase arrest. Thus, these agents significantly decreased the cell population in this phase, (for example, doxorubicin and resveratrol).
iii. G2/M-phase: This is the final phase of cell replication (mitosis). Compounds that interfere with the stabilization or destabilization of microtubular spindles are expected to significantly increase the cell population in this phase, (for example, paclitaxel and vincristine).

##### Assessment of cellular apoptosis

Apoptotic and necrotic cell populations were determined using an Annexin V-FITC- and PI double staining apoptotic detection kit (Abcam Inc., Cambridge Science Park, Cambridge, UK)- coupled with 2 fluorescent channels flow cytometr. After treatment with test compounds for 72 h, 10^6^ of the tumor or normal cells were collected by trypsinization and washed twice with ice-cold PBS (pH 7.4). Then, the cells were incubated in the dark with 0.5 ml of Annexin V-FITC/PI solution for 30 min at room temperature according to the manufacturer’s protocol. After staining, the cells were injected via an ACEA Novocyte™ flow cytometer (ACEA Biosciences Inc., San Diego, CA, USA) and analyzed for their FITC and PI fluorescence signals using FL1 and FL2 signal detectors, respectively (λex/λem 488/530 nm for FITC and λex/λem 535/617 nm for PI). For each sample, 12,000 cells were analyzed. FITC and/or PI positive cells were quantified by quadrant analysis, and resuts were calculated using ACEA NovoExpress™ software (ACEA Biosciences Inc., San Diego, CA, USA)^26–29^.

i. Early apoptotic phase (Q4): This phase refers to cells that initiate the programed cell death process. In this phase, the cell membrane is still intact.
ii. Late apoptotic phase (Q2): This phase refers to cells that initiate the programed cell death process but whose cell membranes are perforated (damaged).
iii. Necrosis phase (Q1): This phase refers to cells that are undergoing nonprogramed cell death process (necrosis) in which the cell membrane is perforated (damaged).
iv. Normal intact cells: In this case, cells are represented by the cell population in Q3 of the well known flow cytometric pattern.

### Statistical analysis

All experiments were performed in triplicates and results were presented as means ± standard deviation. Results of cytotoxicity assay and cell cycle analysis were subjected to Three-way ANOVA test to reveal the effectiveness of *Gandoerma* species, cell line sensitivity and dose-dependent pattern of cell line to extract concentration or cell cycle phases. Moreover, a paired T-test was used for *Ganoderma* species comparison within the same extract concentration and cell line using SPSS software (v25.0, USA).

## Results

### Cytotoxicities using the sulfo-rhodamine B (SRB) assay

**Fig. 2:**
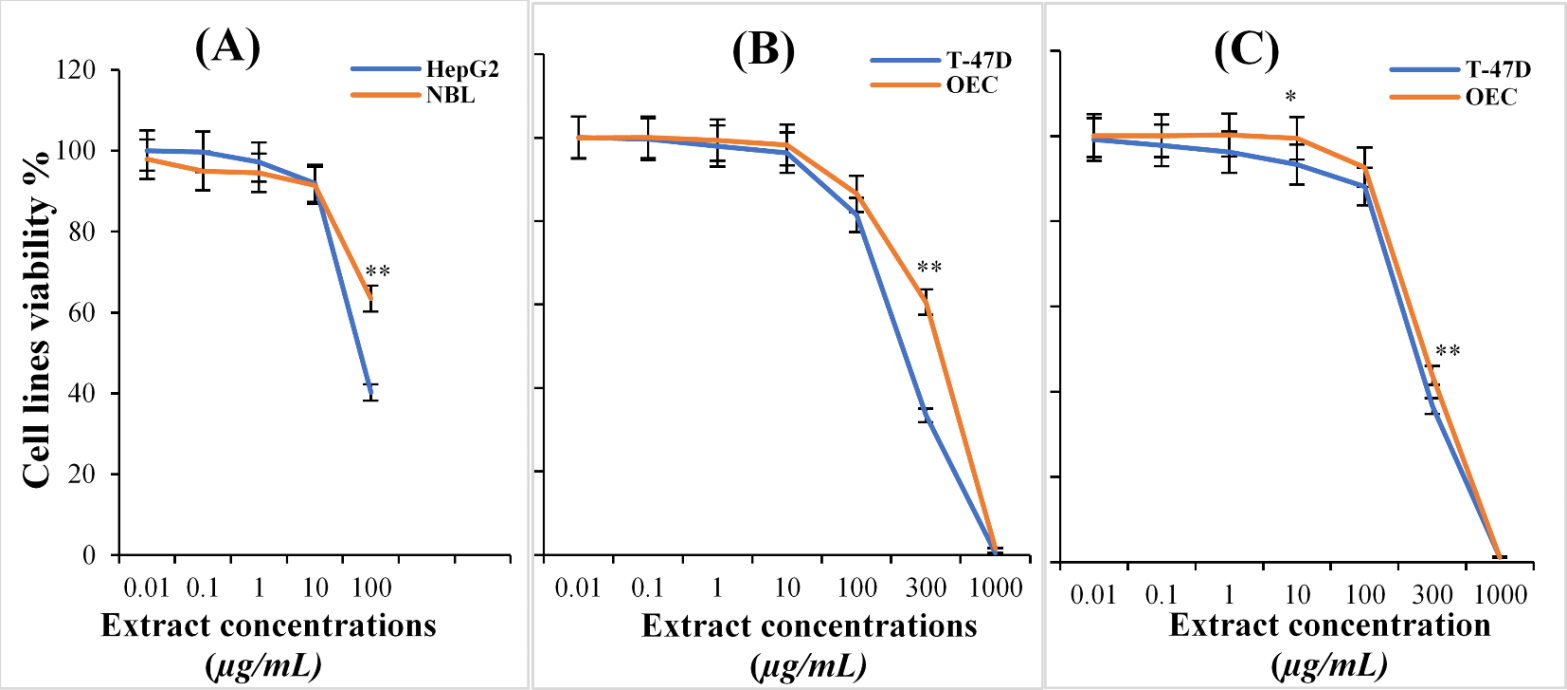
Relative viabilities of different cell lines after incubation with *Ganoderma* extracts for 72 h; HepG2 versus BNL cell lines treated with different concentrations of *G. resinaceum* extract (**A**); T-47D and OEC cell lines treated with different concentrations of *G. australe* (**B**) and *G. mbrekobenum* (**C**) extracts. Bars presenting standard deviation (SD) values of three replicates. (*) on curve means significant difference (*P *< 0.05), and (**) means highly significant difference (*P *< 0.01) between the values of the two cell lines treatments, while absence of these signs means no significant difference between the two values.

The cytotoxicities of different *Ganoderma* species extracts against BNL, HepG2, OEC, and T-47D cell lines were determined using the a bright -pink amino xanthene dye (SRB) binding assay. The results of these cytotoxicities are presented in Fig. 2. *G. resinaceum* extract at concentrations ranging from 0.01 to 100 µg/mL caused a decrease in the cell viability of the of normal cell line (BNL) at an IC_50_ > 100 µg/mL and in the hepatic tumor cell line (HepG2) ; however, at an IC_50_ of 72.32 µg/mL, when using a concentration of 1.0 µg/mL, the cell viability of the normal cell line (BNL) and hepatic tumor cell line (HepG2) were 94.5 ± 1.2 and 97.2 ± 1.3%, respectively On increasing the concentration of *G. resinaceum* extract to reach 100 µg/mL, additional decreases in the cell viability of these two cell lines were observed (63.5 ± 0.9 and 40.3 ± 2.7%, respectively). On the other hand, T-47D tumor cell line and OEC normal cell line were treated with *G. australe* extract at concentrations ranging from 0.01 to 1000 µg/mL for 72 h. showed that the cytotoxicities of *G. australe* extract increased in a dose dependent manner at IC_50_ of 222.0 and 328.3 µg/mL, respectively. At a concentration of 1 µg/mL, the viabilities of these two cell lines, T-47D and OEC slightly decreased to 98.0 ± 1.6 and 99.3 ± 0.5%, respectively. The maximum dose (which was equivalent to 1000 µg/mL) of the *G. australe* extract had the greatest toxic effect on the cell viability of T-47D, which was 0.5 ± 0.4%, while that of OEC was only 1.6 ± 1.2%.

Treatment of the OEC normal cell line and T-47D tumor cell line with *G. mbrekobenum* extracts at concentrations ranging from 0.01 to 1000 µg/mL for 72 h. had low cytotoxicities at an IC_50_ of 271.6 and 236.5 µg/mL, respectively. At a concentration 1 µg/mL of this extract, the cell viability of the OEC and T-47D cell lines was 100.0 ± 0.1 and 96.2 ± 3.2, respectively). Furthermore, the highest dose of *G. mbrekobenum* extract (equivalent to 1000 µg/mL) caused the viabilities of the T-47D and OEC cell lines to be only 1.2 ± 0.7 and 1.2 ± 0.2, respectively. Generally, the results revealed that the extracts were more toxic to the cancer cell lines than to the normal cell lines, especially at higher concentrations.

Results of the Three-way ANOVA for cytotoxicity assay were summarized in Table 2. *Ganoderma* species and cell line showed a significant influence on cell viability (*p* < 0.01), while concentration (0-100) did not show a significant effect independently. Significant interaction effects were observed between *Ganoderma* species and concentration (*p* < 0.001), also between cell line and concentration (*p* < 0.01). However, the interaction between *Ganoderma* species and cell line was not significant. In case of higher concentrations (300–1000) treatments, concentration was the significant affecting factor (*p* < 0.001). Results of T-test analysis showed that only significant differences between tested *Ganoderma* species were found only at extract concentration of 10 ug/ ml (*P* = 0.039) and 300 ug/ ml (as presented in Fig. 2), suggesting that no significant difference for the cell lines at any other concentrations.

**Table 2:** The results of the Three-way ANOVA of cell line viability assay.

Source	Sum of squares	Degree of freedom	F-value	*P*-value
*Ganoderma* species	7144.808	2	3584.05	**4.66 × 10** ^**−7**^
Cell line	73756.95	3	24665.81	**9.86 × 10** ^**−9**^
Concentration	~ 0	4	~ 0	1.00
*Ganoderma* species × Cell line	13.251	6	2.22	0.23
*Ganoderma* species × Concentration	426.960	8	53.54	**8.40 × 10** ^**−4**^
Cell line × Concentration	343.118	12	28.69	**2.67 × 10** ^**−3**^

*P*-values in bold font indicate significance.

### Cell cycle analysis

**Fig. 3:**
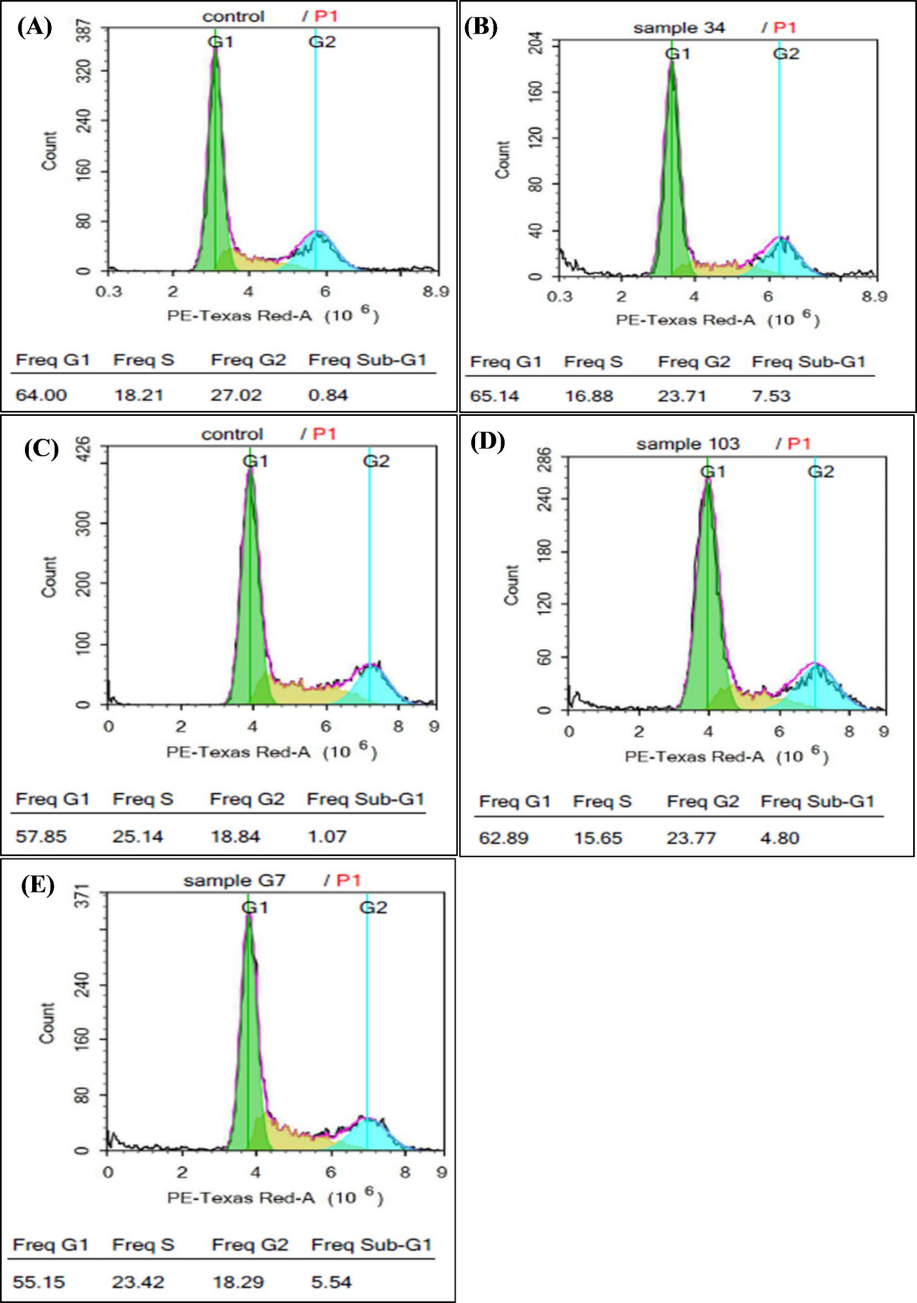
Flow cytometric charts of cell cycle distribution of untreated cell lines and other cells treated with *Ganoderma* extracts: HepG2 untreated control cells (**A**) versus *G. resinaceum* extract treated HepG2 cells (**B**). T-47D untreated control cells (**C**), versus those treated with either *G. australe* extract (**D**) or *G. mbrekobenum* extract (**E**).

T-47D and HepG2 cell lines were used to study the effects of *Ganoderma* extracts on the parameters of these cell cycles in vitro. A flow cytometry protocol was used to assess the population distribution of the different stages of the cell cycle based on the DNA content after staining with PI. Figure 3 shows the flow cytometric charts plotting of these results. After treating the hepatic tumor cell line (HepG2) with *G. resinaceum* extract, most of the cells (55.14%) accumulated in the G1 phase. On the other hand, the percentage of cells in sub G1 phase was only 7.53%, with a reduction in the number of cells in the S and G2 phases (15.9 and 21.7%, respectively).

According to the results shown in Table 3, after treating the T-47D tumor cell line with *G. australe* extracts, the cells accumulated more in the G1 phase than in the G2 phase (59.89 and 21.77%, respectively). On the other hand, the percentages of cells in the S and sub G1 phases were 13.65 and 4.80%, respectively. Similarly, after treating the T-47D cell line with *G. mbrekobenum*, the percentages of cells in the G1, G2, S and sub G1 phases were 54.15, 18.29, 22.42 and 5.54%, respectively.

**Table 3:** Percentages of accumulated cells (T-47D and HepG2) among the different phases of the cell cycle before and after treatment with *Ganoderma* extracts.

Cell cycle phase	Percentages of the accumulated cells %				
	(A) T-47D			(B) HepG2	
	Untreated control	Treated with *G. australe* extract	Treated with *G. mbrekobenum* extract	Untreated control	Treated with *G. resinaceum* extract
G1- phase	56.85 ± 1.36	59.89 ± 1.46	54.15 ± 0.50	54.00 ± 2.90	55.14 ± 1.08
S- phase	24.14 ± 1.62	13.65 ± 1.10	22.42 ± 0.50	18.21 ± 0.34	15.9 ± 0.88
G2- phase	18.84 ± 1.02	21.77 ± 1.62	18.29 ± 0.53	27.02 ± 3.05	21.7 ± 3.01
Sub G1- phase	1.07 ± 0.49	4.80 ± 0.30	5.54 ± 0.28	0.84 ± 0.11	7.53 ± 0.36

Presented values are means ± standard deviation (SD) of three replicates.

Results of the three-way ANOVA of cell cycle analysis were summarized in Table 4. Phase was highly significant (*P* < 0.001), indicating strong differences between cell cycle phases and this is consistently explaining variability in percentages, while neither the *Ganoderma* species, cell line, nor their interactions significantly affected the percentages in any of the models (*P* > 0.05).

**Table 4:** The results of the Three-way ANOVA of cell cycle analysis.

Source	Sum of squares	Degree of freedom	F-value	*P*-value
*Ganoderma* Species	12.93	2	0.01	0.989
Cell line	0.01	1	< 0.01	0.998
Phase	10,044.23	3	223.69	**< 0.001**
*Ganoderma* Species × Cell line	0.98	2	< 0.01	0.999
*Ganoderma* Species × Phase	123.85	6	1.86	0.170
Cell line × Phase	30.45	3	0.68	0.578

*P*-values in bold font indicate significance.

### Apoptotic analysis

**Fig. 4:**
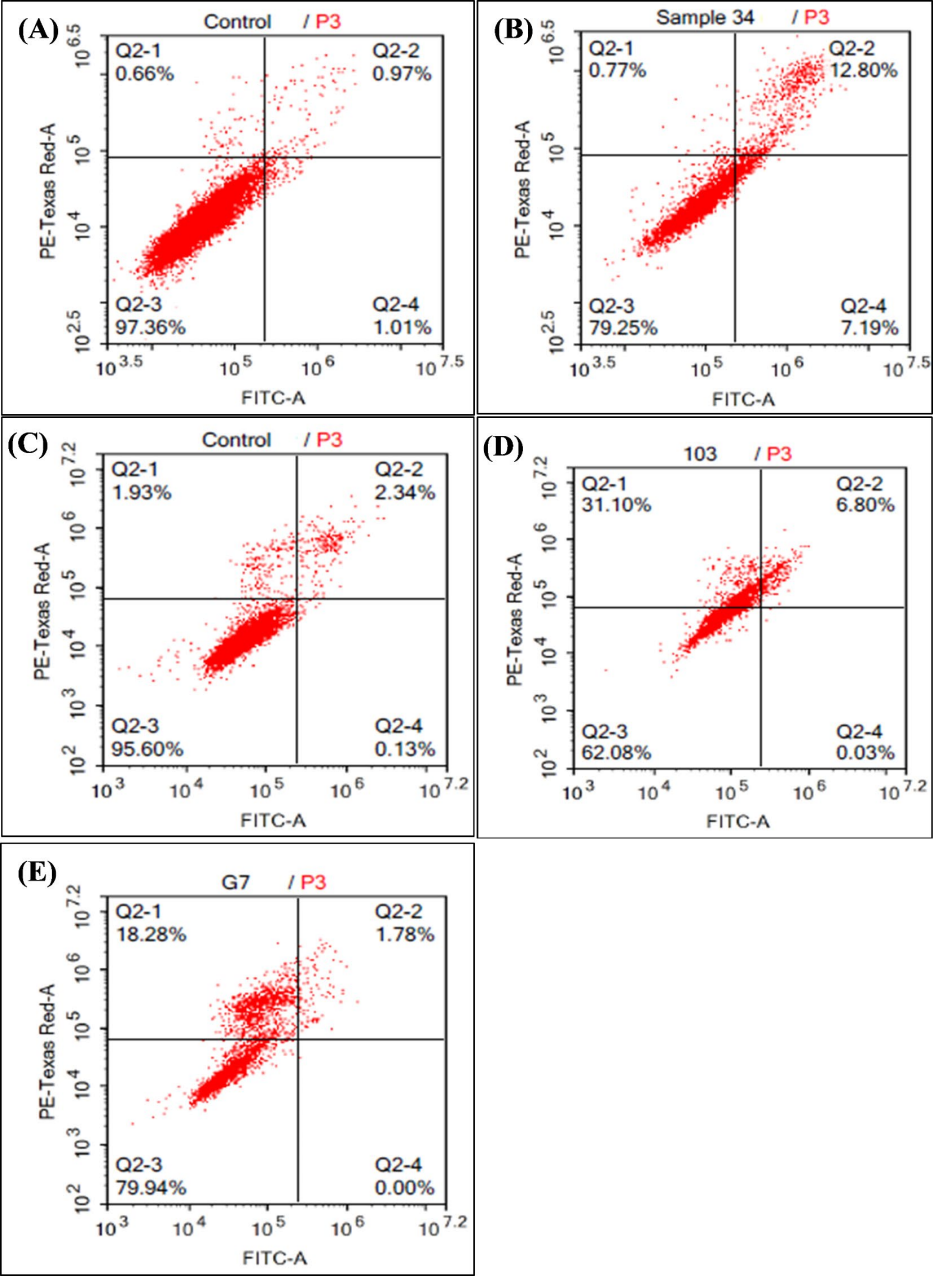
Histograms of apoptotic cells in the untreated and treated cell lines; HepG2 control cells (**A**) versus those treated with *G. resinaceum* (**B**), T-47D control cells (**C**) versus those treated with *G. australe* extract (**D**) or *G. mbrekobenum* extract (**E**).

The percentage of cells undergoing apoptosis induced by treatment with *Ganoderma* species extracts was measured using an annexin V/PI double staining assay and data are presented in Fig. 4. Treatment of the hepatic tumor (HepG2) cell line with *G. resinaceum* increased the total percentage of apoptotic cells (Q2 + Q4) of 19.99% but only 0.77% of necrotic cells (Q1). On the other hand, the percentage of cells that died via necrosis were increased in the T-47D tumor cell line, which was treated with either *G. australe* or *G. mbrekobenum* extracts (31.10 and 18.28%, respectively). Furthermore, only 6.83% and 1.78% of the cells died via the apoptotic mode, respectively.

## Discussion

*Ganoderma* mushrooms are well known for their medicinal activities. The anticancer effects of these compounds are thought to be largely attributed to their diverse chemical constituents^16^. Therefore, this in vitro study was designed to determine whether the methanolic extract of *G. resinaceum* has anticancer effects on the HepG2 tumor cell line. Additionally, this study tested the cytotoxic effects of the methanolic extracts of *G. australe* and *G. mbrekobenum* against the tumor T-47D cell line. Furthermore, the toxic effects of these extracts on the normal cell lines BNL and OEC were also tested.

Our data demonstrated that 100 µg/mL of the methanolic extract of *G. resinaceum* decreased the viability of the HepG2 cell line to 40.3 ± 2.7% at an IC_50_ of 72.32 µg/mL. At the same concentration, the extract decreased the viability of the normal cell line (BNL) to 63.5 ± 0.9%, with an IC_50_ > 100 µg/mL, indicating that the sensitivity of the HepG2 cell line to such an extract was increased by 138.3%. These findings led us to speculate that such an extract is a promising anticancer agent against hepatocellular carcinoma, at least in part on the HepG2 cell line. Liu et al.^30^ reported that, the extracts of *G. lucidum* and *G. sinense* mushrooms inhibited the growth of the HepG2 tumor cell line. However, the antiproliferative effects of the ethanolic extract of *G. lucidum* were significantly greater than those of the *G. sinense* extract. In this regard, the inhibitory effect of *G. lucidum* extract (at a concentration of 120 µg/mL) on HepG2 cells was 39.8%. On the other hand, *G. sinense* extract (at a concentration of 200 µg/mL) inhibited the growth of the same cell line by only 15.5%.

According to the study of Lin et al.^31^, *G. sinense* extract significantly inhibited the growth of HepG2 cells by approximately 60% after 24 h of the treatment at a concentration of 100 µg/mL (IC_50_ 70.14 µg/mL). Moreover, this extract had a slight inhibitory effect on normal liver epithelial cells (HL-7702 cells), with an IC_50_ of 280.56 µg/mL. Chen et al.^32^ tested the anti-proliferative effects of *Ganoderma* extracts and spores’ oil on human chronic myeloid leukemia (K562), human acute myeloid leukemia (HL60), and human gastric carcinoma (SGC-7901) cell lines at concentrations ranging from 0.22 to 1.10 mg/mL. *Ganoderma* extracts had IC_50_ values of 0.44, 0.39, and 0.90 mg/mL for K562, HL60, and SGC-7901 cells, respectively. These IC_50_ values were much greater than our values. MTT assays revealed that *Ganoderma* spores oil concentrations ranging from 0.31 to 10.0 mg/ml caused dose-dependent cytotoxicities in K562, HL60, and SGC-7901 cells, with IC_50_ values ranging from 1.13 to 2.27 and 6.29 mg/mL, respectively.

In the present study, analysis of the cell cycle distribution of the hepatic tumor HepG2 cell line treated with *G. resinaceum* extract showed that most cells (55.14%) accumulated in the G1 phase. Additionally, the percentage of cells in sub G1 was only 7.53%, with a reduction in the number of cells in the S and G2 phases (15.9 and 21.7%, respectively). On the cell cycle level, Lin et al.^31^ tested whether Salubrinal; which is a chemical inhibitor of endoplasmic reticulum (ER) stress caused by *G. sinense* extract, affects the cell cycle phases in HepG2 cells. They found a significant reduction in the proportion of HepG2 cells in the G2/M phase at concentrations of 50 and 100 µg/mL to be 29.1 ± 1.1% and 32.2 ± 1.3%, respectively compared to that in cells treated with *G. sinense* extract alone at the same concentrations (33.7 ± 1.0% and 43.7 ± 1.2%). Their findings indicate that ER stress is an essential part in GSE-induced G2/M phase cell cycle arrest. Thus, one can argue that the antitumor mechanism to the induction of ER stress; in the present study, as they used the same extract in their point of research. This type of stress can participate in the inhibition of essential protein expression which will be followed by inhibition of essential functions necessary for tumor cells or even normal cells growth. As a result, tumor cells growth inhibition is not unexpected^33^.

In this regard, Chen et al.^32^ showed that oil from *Ganoderma* spores (1.25 mg/mL) caused a the G1 phase cell arrest. The percentage of human chronic myeloid leukemia (K562) cells that accumulated in S phase after treatment with this oil was reduced from 55.7 ± 0.6 to 42.3 ± 0.2 after 12 h of treatment. When this concentration was increased to 2.5 mg/mL, the percentage of cells arrested at the S- phase decreased to 38.7 ± 3.2, while the percentage of cells in the G1-phase increased from 32.7 ± 0.5 to 43.7 ± 2.2. Accordingly, the present study showed that *G. resinaceum* stimulated a total apoptotic cell death of 19.99%. on the HepG2 cell line (Q2 + Q4). On the other hand, its necrotic effect was only 0.77% (Q1).

Furthermore, Lau et al.^34^ used hexane and chloroform fractions of *Ganoderma neo-japonicum* extracts to assess their apoptotic effects on a human colonic carcinoma cell line (HCT116). These authors showed that such extracts significantly (*P*˂0.05) increased the proportion of apoptotic cells (41.2 ± 3.5% and 84.4 ± 0.1%, respectively) at a concentration of 100 µg/mL. These ratios were higher than those of our study. Similarly, both fractions increased the rate of apoptosis in the human colonic carcinoma cell line HT29 in a dose-dependent manner. Among the cells, 23.4 ± 3.0% and 44.0 ± 3.0% of the cells were apoptotically populated at the high dose (100 µg/mL) for hexane and chloroform, respectively. Thus, one can conclude that the antitumor effect of *Ganoderma* extracts is solvent dependent not only from the perspective of natural product practitioners but also from the perspectives of GC mass spectrometry and other chromatographic tools^35^.

Our results showed that the *G. australe* extract had a greater cytotoxic effect on the T-47D tumor cell line compared with extract of *G*. *mbrekobenum.* This is because, at 300 µg/mL of *G. australe* extract, the viabilities of the T-47D and OEC cell lines were 33.5 ± 2.6 and 60.6 ± 3.1%, respectively, at IC_50_ values of 222.0 and 328.3 µg/mL, respectively; conversely, at the same concentration of *G. mbrekobenum* extract, the viabilities of the T-47D and OEC cell lines became 36.7 ± 1.6 and 43.8 ± 2.3%, respectively, at IC_50_ values of 236.5 and 271. 6 µg/mL, respectively. Hu et al.^36^ tested the anticancer effect of a water extract of *G. mbrekobenum* mushroom which contains 1.12% polysaccharide. The obtained inhibition results on T-47D cell lines were potentially valuable. In addition, their ethyl acetate extract showed a positive inhibition rate for HepG2 tumor cell line depended on the concentration. The inhibition rates of the concentrations (25, 50 and 100 µg/mL) were (3.4 ± 2.33, 16.2 ± 4.59 and 34.7 ± 2.12%, respectively) with IC_50_ 163.37 ± 18.79 µg/mL. Wongkhieo et al.^37^ investigated the anticancer effect of the mycelia extract of a Thailand’s wild *Ganoderma australe*. The analysis of mycelial extracts active ingredients using a liquid chromatography-tandem mass spectrometry (LC‒MS/MS) method revealed the presence of lovastatin and tentative compounds including p-coumaric, nicotinamide, gamma-aminobutyric acid, choline, nucleosides, amino acids, and saccharides. These extracts had an inhibitory effect on the activity of HMG-CoA reductase in a concentration-dependent manner. At 2.5 mg/mL, the *G. australe* extracts did not interfere with the viability of HepG2 spheroids, but their biochemical composition was altered as determined by Fourier-transform infrared (FTIR) spectroscopy.

Hereher et al.^38^ showed that mushroom polysaccharide (PS), the main active ingredient in mushrooms, inhibits cancer cells by activating host immune responses. In addition, these special properties, in addition to their minimal side effects, potentiate the use of mushroom PSs as novel anticancer agents. Furthermore, these PSs were obtained from the fruiting bodies of *Volvariella speciosa.* The IR spectrum of this extract contains characteristic bands, which are usually attributed to the presence of (1–3) β-glucan linkages with protein moieties. These PSs had antitumor effects on the growth of Ehrlich ascites carcinoma (EAC) cell lines, which were intraperitoneally injected into mice in a pilot study either as a treatment protocol or as a prophylactic agent. The use of prophylactic agents didn’t rule out the antitumor effect of these PSs. However, the treatment mode produced better results. A significant reduction in tumor volume in tumor-bearing mice treated with PS was accompanied by a significant reduction in both liver DNA and RNA. In addition, the mean total lipid and protein levels in liver tissues increased after treatment, indicating clearance of the metastasized tumor in the liver or protection of liver cellular membranes. Moreover, a significant increase in mean superoxide dismutase (SOD) activity and a reduction in the mean malondialdehyde (MDA) concentration were reported to indicate a reduction in oxidative stress. Furthermore, compared with those in saline-treated tumor-bearing mice, highly significant decreases in the serum alkaline phosphatase (ALP), alanine transaminase (ALT) and aspartate transaminase (AST) levels and simultaneous elevation in the serum albumin concentration were observed after mice treatment with the mushroom PS compared with those of the saline-treated tumor-bearing mice. These results confirm the enhancement of liver function after treatment. As expected, the hematological parameters were found to be altered toward normal values. Immunologically, elevations in the mean levels of IFN-γ, IL-2, and IL-12 and a reduction in IL-10 concentration were recorded. Therefore, they concluded that these PS should be used in different potential applications in the future. This may also be the case for the three mushrooms extracts used in this study if the in vivo study was performed. A reduction in tumor cell viability and arrest of cell cycle parameters as well as cellular apoptosis and necrosis, are the main guides for this conclusion.

Zhang and Lin^39^ and Zhang et al.^40^ reported that the addition of *G. lucidum* water extract or its polysaccharides to cultures of murine sarcoma (S-180) or human acute myeloid leukemia (HL-60) tumor cell lines had no inhibitory effect on the proliferation of these tumor cells. Furthermore, these compounds had no apoptotic efficacy, even at very high concentrations of *G. lucidum* polysaccharide (400 mg/L). Furthermore, Hanyu et al.^18^ showed that the inhibition rates of *Ganoderma applanatum* polysaccharide (GAP-3 S) on MCF-7 cell growth were 37.2% and 51.1% after 24 and 48 h of treatment, respectively. The Egyptian *G*. *resinaceum* was previously investigated in El-Sherif et al.^41^ for their anti-breast cancer effect, a clear inhibition was observed in the growth of MCF-7 and MDA-MB-231 breast cancer cell lines in vitro, with IC_50_ values of 1.18 µM and 12.82 µM, respectively. Furthermore, Sedky et al.^42^ assessed the antitumor activity of the phytosterol α-spinasterol isolated from the Egyptian *G. resinaceum* mushroom on human breast cancer cell lines (MCF‐7, MDA‐MB‐231) as well as on a human ovarian cancer cell line (SKOV‐3). In addition, the isolated α‐spinasterol from their mycelial extract showed an antitumor effect on these types of cancer cell lines. These effects were evaluated by MTT cell viability and Annexin V/propidium iodide apoptotic assays as we did. The molecular mechanism underlying this effect was assessed by the relative expression of tumor suppressor genes, namely, p53, BRCA1 and BRCA2, and the apoptotic marker (Bax), a cell cycle progression marker, namely, the cyclin-dependent kinase cdk4/6, which was evaluated using real-time PCR. Moreover, cell cycle analysis was performed for the three investigated cancer cell lines to explore the effects of the presence of α‐spinasterol on cell cycle progression. In this regard, significant increases in the expression of p53 and Bax were observed in cells treated with α-spinasterol, while cdk4/6 were significantly downregulated upon exposure to these sterols. Furthermore, cell cycle analysis of these sterol-treated cells revealed that sterol had a significant inhibitory effect on breast and ovarian cancer cell lines in a time- and dose-dependent manner. These findings support the ability of α-spinasterol to increase p53 and Bax expression and lower cdk4/6 expression.

El-Esseily^35^ had studied the methanolic fraction composition of the Egyptian *Ganoderma* mushrooms; *G. resinaceum* EGM and *G. mbrekobenum* EGDA, revealing a complex mixture of different classes of compounds identified by comparing their GC/MS spectrum with that from the NIST library. The methanolic fraction of *G. resinaceum* EGM consisted of nine different compounds belonging to four polyhydrocarbons and two aliphatic unsaturated fatty acids, while that of *G. mbrekobenum* EGDA composed of 17 different compounds belonging to five amines, one aldehyde, two unsaturated esters, one piperidine, one unsaturated aliphatic hydrocarbon, one aromatic acid, one indole, one ketophenol, one saturated aliphatic hydrocarbon, one saturated aliphatic acid and two unsaturated fatty acids. As unsaturated fatty acids are well known for their antioxidant effects, these antioxidant activity would be responsible for their antitumor effects^43^. Additionally, piperidine is well known for its excellent anticancer properties^44^. The three *Ganoderma* spp. of present study; *G. resinaceum*, *G. australe* and *G. mbrekobenum*, were reported to produce lanostane. Lanostane is a major metabolite of macro fungi that possesses enormous substitution diversity and remarkable biological activities, especially anticancer, antioxidant, and anti-inflammatory effects Wahba et al.^5^, Isaka et al.^45^, Chen et al.^46^.

Smina et al.^47^ studied the triterpene contents of *Ganoderma lucidum* and showed that these compounds had remarkable cytotoxic effects on MCF-7 cells, possibly by inhibiting their proliferation. The extent of increase in the cytotoxicity was concentration and time dependent. In the present study, after treating the T-47D tumor cell line with *G. australe* extract, these cells were more accumulated in the G1 and G2 phases (59.89 and 21.77%, respectively). On the other hand, the percentages of these cells in the S and sub G1 phases decreased by only 13.65 and 4.80%, respectively. The percentages of cells that were registered after treating the T-47D cell line with *G. mbrekobenum* in the G1, G2, S and sub G1 phases were 54.15, 18.29, 22.42 and 5.54%, respectively. Hsu et al.^48^ showed that, after treating of colorectal cancer cells (Colo205) with *Ganoderma tsugae* extract, the proportion of cells in G2/M phase significantly increased with increasing concentrations of this extract. A reduction in the levels of both cyclin A and B1 but not cyclin D or E was observed in these cells. Therefore, they investigated the involvement of cyclin-dependent kinases (CDKs), which promote cell cycle progression, as well as CDK inhibitors, namely, p21 and p27. In their study, treatment of Colo205 cells with *Ganoderma tsugae* extract for 24 h upregulated in the expression of p21 and p27 (CDK inhibitors) and downregulated the expression of cyclin A and B1 (promoters of cell cycle progression). Therefore, these mechanisms may participate in G2/M arrest not only during Colo205 cell killing in Hsu et al.^48^ but also during tumor cell killing in our study. Unfortunately, these markers were not evaluated in our study but their use for identifying *Ganoderma* species is the cause of our invention.

Jiang et al.^49^ showed that treatment of MDA-MB-231 cells, a breast cancer cell line with 0.5 mg/mL of *Ganoderma lucidum* caused cell cycle arrest at the G0/G1 phase. On the other hand, the percentage of MDA-MB-231 cells in G0/G1 increased from 38.7% at the beginning of the experiment to 66.5% and 63.9% after 24 h. and 48 h., respectively. On the other hand, the percentages of T-47D tumor cells that were treated with the *G. australe* and *G. mbrekobenum* extracts and died via necrosis increased (31.10 and 18.28%, respectively). Conversely, only 6.83% and 1.78% of the cells died via the apoptotic mode, respectively. Wu et al.^50^ treated the human pancreatic cancer cell lines PANC-1 and Mia PaCa-2 with Fudan-Yueyang *Ganoderma lucidum* (FYGL) and *Ganoderma lucidum* fruiting body extract and reported that such an extract caused up to a 23% increase in the apoptotic rate of PANC-1 cells during death at 500 µg/mL.

In conclusion, *G. resinaceum* had a better cytotoxic effect on the hepatic tumor cell line HepG2 at an IC_50_ of 72.32 µg/mL than on the normal cell line (BNL) at an IC_50_ > 100 µg/mL. On the other hand, the *G. australe* extract had a better cytotoxic effect on the T-47D tumor cell line compared with extract of *G*. *mbrekobenum* at the respective IC_50_ values of 236.45 and 271. 56 µg/mL. After treatment with *G. resinaceum* extract, most of the cells (55.14%) accumulated in the G1 phase. Furthermore, after treatment with *G. australe* and *G. mbrekobenum* extracts. For the *G. australe* extract, the proportions of cells in the G1 and G2 phases were 59.89 and 21.77%, respectively. after treatment with *G. australe*. The number of cells that were registered after treatment with *G. mbrekobenum* in the sub G1 phase was 5.54%. *G. resinaceum* stimulated a total apoptotic cell death (Q2 + Q4) of 19.99%; on the other hand, the *G. australe* and *G. mbrekobenum* extracts stimulated a percentage of cell death via necrosis, which was 31.10 and 18.28%, respectively). Thus, *G. resinaceum* significantly inhibited the viability of the HepG2 cell line, while both the *G. australe* and *G. mbrekobenum* extracts significantly decreased the viability of the T-47D cell line. These results may encourage their possible use for therapeutic management of hepatocellular carcinoma and breast ductal carcinoma after further in vitro and in vivo investigations. Additionally, their differential targeting of tumor cells compared to normal cells lead one to hypothesize that targeted delivery of the extracts using smart nanocarriers with dual acid and thermal responsiveness, a well-known criterion of the tumor microenvironment, will be beneficial in the future but, further in vivo investigations are needed.

## Data Availability

The datasets used and analysed during the current study are available from the corresponding author on reasonable request.
